# Effectiveness of Virtual Reality on Functional Performance after Spinal Cord Injury: A Systematic Review and Meta-Analysis of Randomized Controlled Trials

**DOI:** 10.3390/jcm9072065

**Published:** 2020-07-01

**Authors:** Amaranta De Miguel-Rubio, M. Dolores Rubio, Alejandro Salazar, Rocio Camacho, David Lucena-Anton

**Affiliations:** 1 Department of Nursing, Pharmacology and Physiotherapy, University of Cordoba, 14004 Cordoba, Spain; z42mirua@uco.es; 2 Department of Cell Biology, Physiology, and Immunology, University of Cordoba, 14007 Cordoba, Spain; ba1rulum@uco.es (M.D.R.); m92caagr@uco.es (R.C.); 3 Department of Statistics and Operational Research, University of Cadiz, 11009 Cadiz, Spain; 4 Institute of Research and Innovation in Biomedical Sciences of the Province of Cadiz (INiBICA), University of Cadiz, 11009 Cadiz, Spain; 5 The Observatory of Pain, University of Cadiz, 11009 Cadiz, Spain; 6 Department of Nursing and Physiotherapy, University of Cadiz, 11009 Cadiz, Spain; david.lucena@uca.es

**Keywords:** virtual reality, neurological rehabilitation, spinal cord injuries, physical therapy, functional performance, quality of life

## Abstract

A spinal cord injury (SCI) usually results in a significant limitation in the functional outcomes, implying a challenge to the performance of activities of daily living. The main aim of this study is to analyze the effectiveness of virtual reality to improve functional performance in patients with SCI. The search was performed between October and December 2019 in Cumulative Index to Nursing and Allied Health Literature (CINAHL), Medline, Cochrane Central Register of Controlled Trials, Physiotherapy Evidence Database (PEDro), PubMed, Scopus, Web of Science, and Embase. The methodological quality of the studies was evaluated through the PEDro scale, and the risk of bias was evaluated with the Cochrane collaboration’s tool. Seven articles were included in this systematic review, and five of them in the meta-analysis. Statistical analysis showed favorable results for functional performance in control group performing conventional therapy, measured by the functional independence measure (standardized mean difference (SMD)= −0.70; 95% confidence interval: −1.25 to −0.15). Results were inconclusive for other outcomes. Most studies have not shown beneficial effects on functional performance compared with conventional physical therapy. The results obtained showed that virtual reality may not be more effective than conventional physical therapy in improving functional performance in patients with SCI.

## 1. Introduction

A spinal cord injury (SCI) affects the conduction of sensorimotor signals, causing temporary or permanent alterations [1] on mobility or autonomic function below the level of the injury, so that the more cranial the injury, the more severe it is. This leads to a significant limitation in the functional outcomes and patient’s activities of daily living (ADLs), and a loss of quality of life [2,3]. Increased survival after traumatic SCI has resulted in an increase in its prevalence over the past 20 years. Furthermore, people with physical disability produce a high impact on the health system and a higher burden for society [3].

Neurorehabilitation involves a set of methods or techniques that aim to maintain or recover lost or decreased neurological functions as a result of brain or spinal damage [4]. In addition to conventional physical therapy (CPT), new rehabilitation tools such as virtual reality (VR) have emerged in recent years. Today, VR represents a multidisciplinary tool in clinical medicine, which is used in many applications including pain management, assessment of neurocognitive impairment, training of medical techniques and physical rehabilitation [5]. CPT has been shown to be monotonous for patients, as they tend to be asked to perform the same gesture or activity during treatment [6]. Alternatively, in recent years there has been a growing interest in the use of VR, video games or even video games that include physical therapy (exergaming) [7].

VR can be defined as a simulation of a real environment generated by a computer in which the subject can interact with certain elements within a simulated space through a human-–machine interface [2]. These systems provide the possibility of recreating safe virtual scenarios for practicing activities that in the real world would entail a potential risk, developing telerehabilitation platforms, monitoring patients based on the data recorded, accurately controlling each session, objectively evaluating the execution of the therapy and providing incentives for the patient to ensure adherence to the treatment [7]. VR interventions are usually applied through videogames as a therapeutic option, since they are considered more fun than CPT, which motivates the patient to not abandon rehabilitation [6]. One of the advantages of this system is that sports, recreation and functional activities can be performed without any risk [8]. One example of a system that has become popular at all ages in recent years is the Nintendo Wii Fit [9]. The Wii Fit system consists of a balance board that is similar to a force platform. The system helps people with a severe functional disability to become more independent. This type of technology has the advantage that rehabilitation can be done not only in hospitals but also at home [10]. To measure the effectiveness of such techniques, an evaluation using clinical and functional scales was performed before and after the treatment program to identify motor and functional recovery. Two of the most commonly used functional assessments for patients with quadriplegia are the functional independence measure (FIM) and the spinal cord independence measure II (SCIM II). These tests are valid and reliable, and a strong correlation between them has been shown [11].

Due to the rise of this novel technology application in clinical neurorehabilitation in recent years [12], a large number of studies on the use of VR interventions have been conducted in different neurologic disorders, such as: cerebral palsy [13,14], multiple sclerosis [15,16,17], stroke [18,19,20,21], Parkinson’s disease [22,23]. However, the ratio of functional recovery is different for each disease, so the desired effects provided by VR interventions could be different. It must be considered that patients with SCI suffer a great decrease of participation [24] and less than one percent of patients with SCI acquire a full functional recovery at discharge [25], so the neurorehabilitation should be focused on maintaining the remaining functionality after SCI and even low improvements provoked by additional interventions to usual care could imply significant benefits in patients with an SCI [26].

Different recent reviews analyzed the potential use of VR-based neurorehabilitation in patients with SCI. The systematic review conducted by de Araújo et al. [27] concluded that VR therapy could be effective in improving aerobic function, balance, pain level and motor function, but the review was not restricted specifically to assess the quality of life through controlled trials. Villiger et al. [28] suggested that VR interventions may be useful as a neurorehabilitation tool to improve motor function in subjects with a chronic SCI, but authors only analyzed the effects provided by home-based VR interventions. A structured review carried out by Yeo et al. [25] showed positive effects of VR interventions after SCI related mainly to posture and balance, but they focused on the effects on mobility. Finally, Kloosterman et al. [29] also discussed the virtues of VR therapy to enhance motor learning in patients with SCI due to the facilities to control and change the exercise variables, but they did not analyze specifically the effects of VR interventions. Nevertheless, the current evidence through meta-analyses analyzing the use of VR in patients with SCI is limited. Therefore, this systematic review and meta-analysis of randomized and non-randomized controlled trials aimed to evaluate the effectiveness of VR interventions on functional performance in patients with SCI.

## 2. Materials and Methods

### 2.1. Search Strategy

This review was performed according to the PRISMA (Preferred Reporting Items for Systematic Reviews and Meta-Analyses) [30] guidelines and it was registered in the PROSPERO database of prospectively registered systematic reviews (CRD 42018093855). The scientific search was carried out between October and December 2019 in the following electronic databases: CINAHL, Medline, Cochrane Central Register of Controlled Trials, Physiotherapy Evidence Database (PEDro), PubMed, Scopus, Web of Science, and Embase. The following descriptor terms combined with Boolean operators were employed: (“spinal cord injury” OR “spinal cord injuries” OR “paraplegia” OR “quadriplegia” OR “tetraplegia”) AND (“virtual reality” OR “virtual reality exposure therapy” OR “virtual systems” OR “augmented reality” OR “videogame” OR “video games” OR “exergames” OR “exergaming” OR “play-based therapy” OR “commercial games”). In PubMed, Medical Subjects Headings (MeSH) descriptors were used: “virtual reality”, “virtual reality exposure therapy”, “video games”, and “spinal cord injuries”. The search was restricted to clinical trials published as full-text articles and proceeding full-text papers. No language and date filters were applied.

### 2.2. Selection Criteria

The selection criteria were established according to the PICO (Participants, Intervention, Control, and Outcomes) strategy: (1) population: adults with SCI; (2) intervention: game-based interventions through VR; (3) comparison: group performing CPT; (4) outcome: outcomes specifically related to functional performance. Only controlled clinical trials were included. Articles were excluded when: (1) participants were people with and without SCI, but the outcome data were not available for each specific population; (2) control group was performed by health subjects.

### 2.3. Study Selection Process and Data Extraction

This systematic search was performed by combining keywords in different scientific databases. After that, we excluded duplicated articles. Subsequently, titles and abstracts were reviewed, and those articles that did not meet the proposed selection criteria were excluded. The remaining articles were accurately evaluated. In addition, the reference lists of all the identified articles were analyzed for potential additional studies. After excluding those that did not meet the inclusion criteria, the studies obtained were finally included in the systematic review. Two reviewers (A.M.R. and M.D.R.L.) participated independently in the study selection process, review and systematic data extraction. A third reviewer (D.L.A.) participated in achieving consensus in case of controversy.

For each study, the following data were extracted: (1) author and date of publication; (2) number and age of participants, levels of injury and time since onset of SCI; and (3) characteristics of the interventions (type of intervention in each group, outcome measures, and measuring instrument) and results.

### 2.4. Assessment of the Methodological Quality of the Studies Included in the Review

The PEDro [31] scale was used to evaluate the methodological quality of the studies. This consists of 11 items related to the domains of selection, performance, detection, information, and attribution bases. Each item is scored with 1one point if the study meets the criteria, except for criterion number 1. A higher score shows a higher methodological quality. A study with a PEDro score of 6 or higher is considered as a high level of methodological quality (6–8: good; 9–10: excellent), and a study with a score of 5 or less is considered as low level of methodological quality (4–5: acceptable; <4: poor) [32].

### 2.5. Assessment of the Risk of Bias of the Studies Included in the Review

The risk of bias assessment was conducted using The Cochrane collaboration’s tool [33], through Review Manager 5.3 software, which includes a description and evaluation of each item by means of a bias table. This evaluation includes different questions about the risk of bias of the studies and is categorized as: “low risk”, “high risk” and “unclear risk”. Two assessors carried out the evaluation independently after reading the original texts. Then, when there was a difference in the scores between the assessors, the final score was determined through discussion including a third assessor.

### 2.6. Statistical Analysis

For the meta-analyses, the studies were separated into subgroups according to the measuring instrument used. A study could be included in more than one subgroup if it used more than one instrument. In all cases, the groups compared were CPT versus VR interventions. The differences in the effect size (post-pre intervention) between the groups were analyzed in terms of the standardized mean difference, with 95% confidence interval. We set the significance level at *p* < 0.05.

The heterogeneity in each subgroup was determined by the chi-square test and the I^2^ statistic. A fixed-effects model was used in the subgroups where homogeneity was observed. Random-effects models were used in the case of heterogeneity.

The analyses were carried out in Review Manager (RevMan) 5.3 (the Cochrane Collaboration, the Nordic Cochrane Centre, København, Denmark), and the results are presented in tables, including the forest plots on the right.

## 3. Results

**Figure 1:**
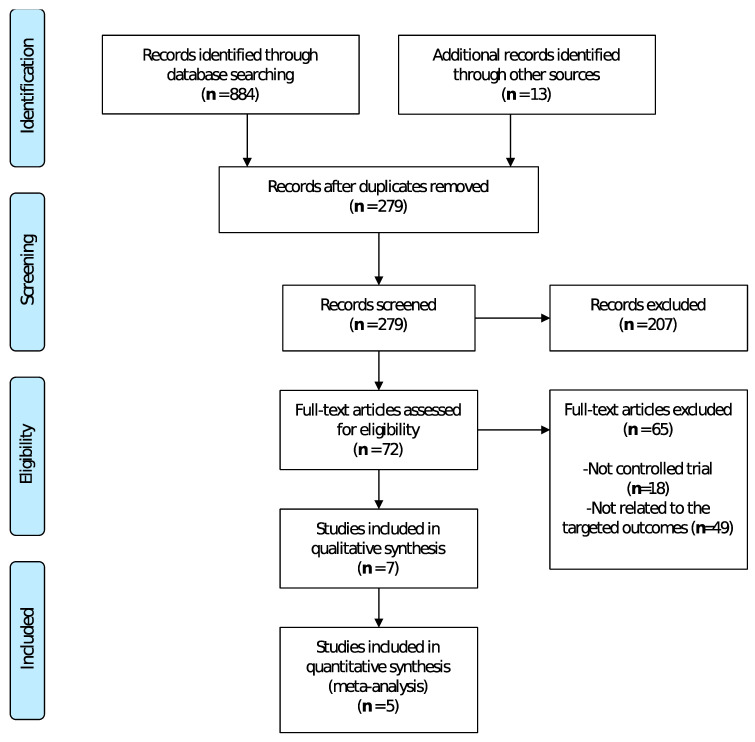
Flow diagram of the different phases of the systematic review and meta-analysis.

The selection process of this systematic review and meta-analysis is shown in Figure 1, retrieving a total of 279 potentially relevant articles. A total of seven studies were included in the systematic review, and five of them in the meta-analysis.

### 3.1. Assessment of the Methodological Quality of the Studies Included in the Review

The scores achieved in the PEDro scale are shown in Table 1. Six studies had high methodological quality with PEDro scores ≥6: Gil-Agudo et al. [7], D’Addio et al. [6], Dimbwadyo-Terrer et al. (2016) (a) [2], Dimbwadyo-Terrer et al. (2016) (b) [34], Khurana et al. [35], and Prasad et al. [36]. Dimbwadyo-Terrer et al. (2013) [11] scored 5, achieving the lowest score.

### 3.2. Assessment of the Risk of Bias of the Studies Included in the Review

**Figure 2:**
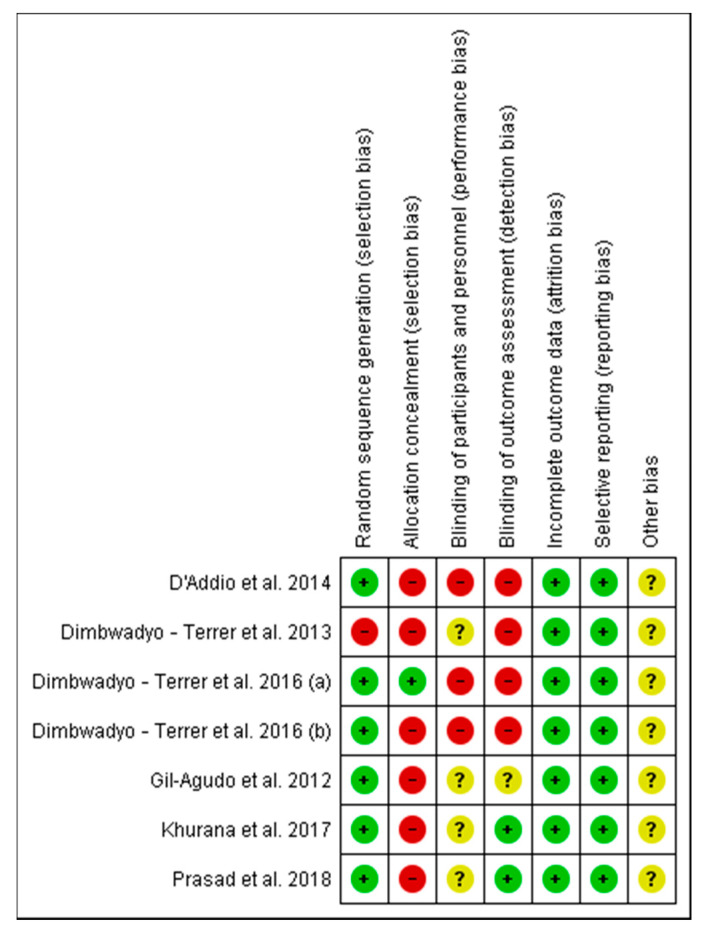
Risk of bias of the studies included in the systematic review.

**Figure 3:**
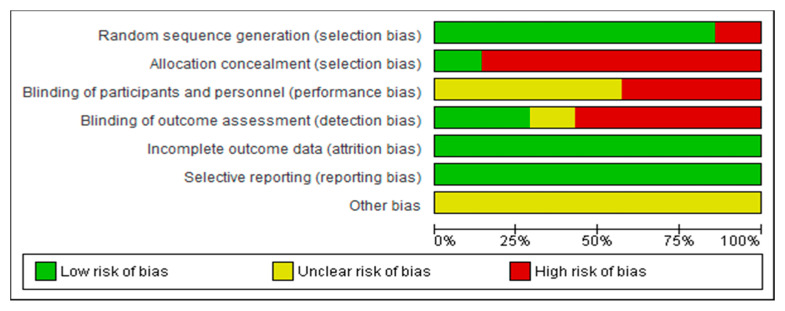
Overall risk of bias. Each category is presented by percentages.

Concerning the assessment of the risk of bias for each of the studies included in this review, the researches carried out by Dimbwadyo-Terrer et al. 2016 (a) [2], Khurana et al. [35] and Prasad et al. [36] had the lowest risk of bias, as shown in Figure 2. Likewise, regarding the risk of bias among all the included studies, the lowest biases are presented in the incomplete outcome data (0%) and the selective reporting (0%), while the highest percentage (85.5%) was obtained in the allocation concealment, as shown in Figure 3.

### 3.3. Data Extraction

A total of 150 participants (control group (CG), *n* = 69; intervention group (IG), *n* = 81) were included in the analyzed studies. Regarding participant age, the highest mean age among the CG belonged to the study by Gil-Agudo et al. [7], with 49.0 ± 6,11 years, whereas the highest among the IG belonged to the study by Dimbwadyo-Terrer et al. (b) [34], with 54.3 ± 9.86 years. The lowest ages for CG and IG appeared in the study by Prasad et al. [36], with 33.9 ± 7.1 and 23.7 ± 5.2 years, respectively. Concerning the number of participants, the study by Dinbwadyo-Terrer et al. (a) [2] had the highest number of participants (n = 31). The studies of D’Addio et al. [6] and Khurana et al. [35] included 30 participants. The lowest sample size was achieved by Dimbwadyo-Terrer et al. (b) [34], with 9 participants. Finally, regarding the neurological level of injury, three studies [2,7,35] included participants with the American spinal injury association impairment scale (ASIA) A–B levels, three studies [11,34,36] included ASIA A–D levels, and one study [6] included ASIA C–D levels. Table 2 shows the main characteristics of the participants.

Regarding the VR devices used in the interventions, three studies [2,7,11] used the Toyra^®^ system (National Paraplegics Hospital in Toledo and Rafael del Pino Foundation, Spain). This system contains motion capture elements that reproduce the patient movements in real time and they are displayed through an avatar on the screen. There are different objects in the virtual environment and patients have to interact with them [7]. Two studies [6,36] used commercial video games, supported by Nintendo Wii (Foxconn, Taiwan); specifically D’Addio et al. [6] used Wii Fit with balance board, and Prasad et al. [36] used the Wii Sports Resort (Nintendo Entertainment Analysis & Development Division, Japan) game. The study by Khurana et al. [35] used Sony Play Station 2 (Sony Corporation, Japan) and Eye Toy (Logitech, Switzerland) with three different virtual environments, which were adapted for rehabilitation purposes. Finally, the study by Dimbwadyo-Terrer et al. (2016) (b) [34] used a data glove to interact with the virtual environment in which patients could see their hands while they manipulated objects in real time.

Concerning the VR protocols, the study performed by D’Addio et al. [6] had the longest total duration of intervention (3 times a week for 12 weeks). Regarding the program intensity, the study by Khurana et al. [35] should be noted: they carried out their VR interventions 5 times a week for 4 weeks. The study with the shortest intervention time and program intensity was that of Bimbwadyo-Terrer et al. (2016) (b) [34], who only performed 4 sessions (2 times a week for 2 weeks). Regarding the duration of the sessions, the study by Prasad et al. [36] had the longest session duration (60 min).

With regard to the effects of the different VR-based interventions on specific deficits treated, most studies analyzed the effects on upper limb motor function [2,7,11,34,36]. Other authors focused their interventions on improving upper limb range of motion [7,11], balance [6,35], upper limb strength [7], upper limb dexterity [36], and posture [6]. Most studies reported no significant effects in the different outcomes analyzed. It should be noted that the studies of D’Addio et al. [6] and Khurana et al. [35] showed significant results in balance. Furthermore, significant results were found in posture [6] and muscle strength [11]. Finally, all studies focused their interventions on improving the functional performance of patients with SCI. Table 3 shows the main characteristics of the interventions carried out in the different studies.

### 3.4. Study Groups Included in the Meta-Analysis

A total of five studies were included in the meta-analysis. Different instruments were used to assess the functional performance: functional independence measure (FIM) [37], spinal cord independence measure (SCIM) [38] and its self-care subscale, and the Barthel Index (BI) [39]. These instruments are commonly used to evaluate the functional status in patients with SCI [11]. 

**Figure 4:**
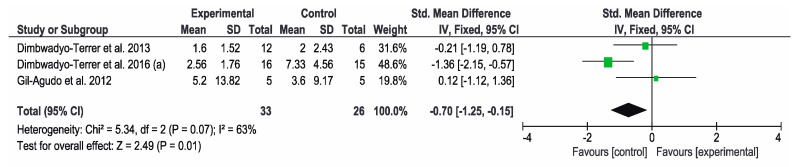
Forest plot for functional performance measured by FIM scale. A green block indicates the weight assigned to the study and the horizontal line depicts the confidence interval. A black rhombus shows the overall result.

Regarding the FIM scale, three studies [2,7,11] analyzed their results on the functional status of the patients. The results showed that CPT resulted in significant improvements compared to VR interventions. The study by Dimbwadyo-Terret et al. (2016) (a) [2] obtained the best results. The overall result of the meta-analysis was favorable to the control group, as shown in Figure 4.

**Figure 5:**
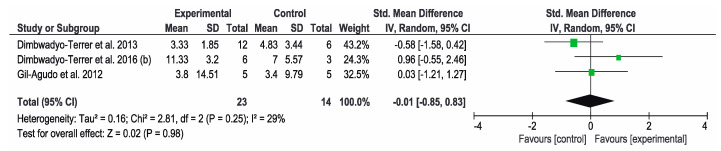
Forest plot for functional performance measured by SCIM scale. A green block indicates the weight assigned to the study and the horizontal line depicts the confidence interval. A black rhombus shows the overall result.

**Figure 6:**
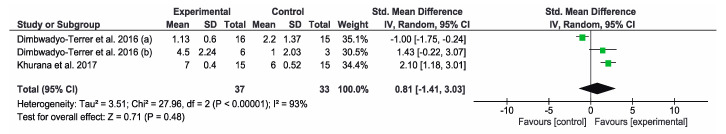
Forest plot for functional performance measured by SCIM self-care subtest. A green block indicates the weight assigned to the study and the horizontal line depicts the confidence interval. A black rhombus shows the overall result.

Concerning the results obtained in the SCIM, the other three studies [7,11,34] used this instrument to assess the functional status. The overall result of this meta-analysis was not conclusive. Favorable results for the control group were obtained in the study by Dimbwadyo-Terrer et al. (2013) [11], while favorable results for the intervention group were obtained in the study by Dimbwadyo-Terrer et al. (2016) (b) [34]. However, none of these results were statistically significant. SCIM self-care subtest was also used to assess the functional performance in three studies [2,34,35]. The overall result of this meta-analysis was not conclusive. Favorable results for the control group were obtained in the study by Dimbwadyo-Terrer et al. (2016) (a) [2], while favorable results for the intervention group were obtained in the study by Khurana et al. [35]. Only the results of Khurana were statistically significant. Figure 5 and Figure 6 show the results of the meta-analysis.

**Figure 7:**
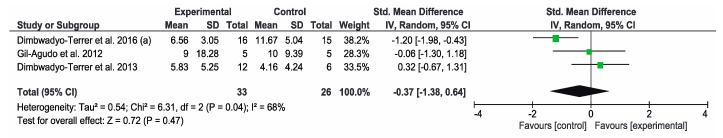
Forest plot for functional performance measured by Barthel index. A green block indicates the weight assigned to the study and the horizontal line depicts the confidence interval. A black rhombus shows the overall result.

Finally, the BI was used to measure the functional status in three of the studies [2,7,11]. The overall result of the meta-analysis was not conclusive and control groups got better results than intervention groups. The study by Dimbwadyo-Terrer et al. (2016) (a) [2] obtained the best results for the control group that carried out CPT. Figure 7 shows the results of the meta-analysis.

## 4. Discussion

This systematic review and meta-analysis aimed to analyze the effectiveness of VR on functional performance in patients with SCI. Seven controlled trials analyzing the effects of different VR interventions compared with CPT were included in the systematic review. These studies used VR systems based on different technological devices, such as Nintendo Wii [6,36], Toyra^®^ system [2,7,11], CiberTouch^™^ data glove [34] and Sony Play Station 2 with Eye Toy [35].

Although VR-based systems could provide many advantages in neurorehabilitation, such as offering precise measurement, increasing motivation, providing direct feedback and safe environments [12,40], the results obtained in our study revealed that VR interventions might not be more effective than CPT in improving functional performance in patients with SCI. Moreover, the statistical analysis showed favorable results of CPT on the functional independence measured by the FIM scale. Our results match with the findings of de Araújo et al. [27], who reported no solid conclusions about the efficacy of VR interventions on quality of life. The authors reported that this can be attributed to the lack of methodological quality and statistical power observed. These results do not match with those of Yeo et al. [25], who showed favorable effects of VR interventions on balance, gait, lower limb motor function and muscle strength. However, the authors highlight the limited quality and scope of the included studies, and seven of the nine reviewed articles were case series. Masseti et al. [41] also reported the potential use of VR in neurorehabilitation, obtaining benefits on motor function, but only two studies included patients with SCI.

Concerning the different technological devices used in the studies, all of them carried out the VR interventions through semi-immersive or non-immersive systems, where a computer or game console projects the virtual environments onto screen displays [42]. We suggest that the inconclusive results on functional performance revealed in the present review could be influenced by this fact, since immersive VR systems were not used in the VR intervention protocols and these VR devices could enhance the task-focused attention [43]. Furthermore, other factors involved in movement generation could influence the results obtained, such as the heterogeneity in terms of protocols carried out, the different tasks performed in the VR sessions, and the different characteristics of the participants. Consequently, this makes it necessary to unify protocols in order to clarify which of the VR devices are more appropriate to obtain the desired effects. Immersive VR devices are more expensive and may need an adequate training to use [41], and they also need further development in order to integrate this technology into the clinical neurorehabilitation [44]. These systems allow ADLs to be practiced in safe virtual scenarios, to optimize motor learning [45], and even to assess and measure the different motor conditions [46]. Therefore, VR devices could be a promising tool in clinical settings for the rehabilitation of patients with neurological disorders. However, according to Morone et al. [47], the effectiveness of VR in different contexts needs to be demonstrated, and precise user guidelines are required before new VR systems becoming commercially available.

It should be noted that only the studies by D’Addio et al. [6] and Khurana et al. [35] showed favorable results on functional performance, measured by SCIM and SCIM self-care, respectively. Incidentally, both studies obtained significant results on static and dynamic balance. Thus, we can hypothesize that the improvements obtained in the functional performance are caused by the improvements obtained in balance, since balance recovery and functional abilities are positively correlated [48]. This correlation was also shown in the study by Prasad et al. [36], who obtained no significant results on balance and functional performance. Moreover, according to the International Classification of Functioning, Disability and Health (ICF) [49], activity limitations can be influenced by impairments at the functional level and by body structure. Consequently, balance impairments could influence the loss of functional performance and vice versa.

Five [2,7,11,34,36] of the seven reviewed articles obtained no significant differences between groups after intervention. It is noteworthy that the study by Dimbwadyo-Terrer et al. (2016) (a) [2], which achieved the highest sample size (*n* = 31), reported better results of the CPT group on functional performance measured by FIM, SCIM self-care and BI. The authors stated that the VR intervention, in addition to CPT, produces similar results to CPT, and they attributed the negative results to the short intervention period. Nevertheless, most of the studies reported high levels of patient satisfaction.

Regarding the intervention and session duration, it should be noted that the studies by D’Addio et al. [6] and Khurana et al. [35] obtained significant results on balance and functional performance. Coincidentally, both studies used the longest intervention durations. Therefore, according to Villiger et al. [50], we can hypothesize that longer training times can produce better effects on functional performance. Consequently, intervention duration could be a key factor in functional recovery after SCI.

Furthermore, other factors related to the design of the studies could influence the results obtained. Patients with SCI could have heterogeneous characteristics depending on the ASIA and injury levels. Regarding the injury severity measured by ASIA levels, three studies [2,7,35] included participants with ASIA A–B levels, three studies [11,34,36] included ASIA A–D levels, and one study [6] included ASIA C–D levels. It is worth noting that the study by Khurana et al. [35], obtained significant results on balance and functional performance in patients with A–B levels. This could be because the patients had low levels of injury (T6–T12) and they had the ability to sit unsupported for at least 10 s and had a minimum of active 90° of shoulder flexion, which can result in greater abilities to enhance functional performance. Another aspect to highlight is that the studies [2,7,11,36] including patients with cervical SCI obtained no significant differences between groups. Therefore, we can state that the recovery on functional performance is related to the level of injury.

Some limitations need to be addressed. One limitation was related to the different injury levels of the patients, since they were not analyzed separately. For this purpose, we encourage authors to use large sample sizes in order to analyze an adequate number of subjects in each stratified group. It could be helpful to know which factors of the participants could affect the results. However, it is difficult in many cases to obtain a higher number of patients, since these patients are treated in a real clinical scenario in conjunction with their prescribed treatment in different centers or institutions. Therefore, most studies use convenience samples, which could result in possible selection biases [51]. Another limitation was the limited number of studies reviewed, so the results should be interpreted with caution.

The present meta-analysis could have clinical implications to bear in mind in future research. We can observe that the non-immersive VR interventions could not produce benefits for functional performance in patients with SCI, so we encourage the use of immersive VR devices in order to encourage the patient’s attention and consequently to achieve better results. In addition, the intervention duration and the injury level could be key factors, so we aim to explore the effects of long-duration VR-based interventions and to determine the VR feasibility according to the injury level, since low levels appear to be more suited to VR interventions. Finally, we also recommend that the effectiveness of the different CPT techniques be investigated, with a view to providing further evidence of their application in neurological rehabilitation.

In view of the above, some additional recommendations for future studies can be drawn. First of all, it would be desirable to unify protocols, as mentioned before, in order to avoid heterogeneity and facilitate the replication by future studies. In addition, studies with higher methodological quality would be recommended, such as multi-centric studies (with larger sample sizes) and/or randomized controlled trials. We encourage researchers to perform these kinds of studies, focusing on the identification of the specific elements of VR interventions that have a greater weight in achieving a positive outcome on functional performance after SCI.

## 5. Conclusions

According to the results presented in our review, we can conclude that the current evidence of VR interventions to improve functional performance after SCI is limited and VR may not be more effective than CPT in improving functional performance in patients with SCI. Furthermore, CPT interventions showed positive effects on functional independence.

Based on our findings, we encourage researchers to perform high-quality clinical trials using larger sample sizes and greater homogeneity in terms of the levels of SCI, devices used and intervention protocols, as well as trying to identify which specific elements of VR interventions could have a greater weight in achieving a positive outcome on functional performance after SCI. In addition, we emphasize the need for clinical trials that prove the effectiveness of the different CPT techniques, in order to provide a deeper knowledge and greater scientific support in the rehabilitation of patients with SCI.

## Figures and Tables

**Table 1:** Scores obtained after methodological evaluation according to the Physiotherapy Evidence Database (PEDro) scale.

Study	1	2	3	4	5	6	7	8	9	10	11	Total
Gil-Agudo et al. 2012 [7]		Yes	No	Yes	No	No	No	Yes	Yes	Yes	Yes	6
Dimbwadyo-Terrer et al. 2013 [11]	-	No	No	Yes	No	No	No	Yes	Yes	Yes	Yes	5
D’Addio et al. 2014 [6]	-	Yes	No	Yes	No	No	No	Yes	Yes	Yes	Yes	6
Dimbwadyo-Terrer et al. 2016 (a) [2]	-	Yes	Yes	Yes	No	No	No	Yes	Yes	Yes	Yes	7
Dimbwadyo-Terrer et al. 2016 (b) [34]	-	Yes	No	Yes	No	No	No	Yes	Yes	Yes	Yes	6
Khurana et al. 2017 [35]	-	Yes	No	Yes	No	Yes	Yes	Yes	Yes	Yes	Yes	8
Prasad et al. 2018 [36]	-	Yes	No	Yes	No	No	Yes	Yes	Yes	Yes	Yes	7

Range: 0–10. Item 1 is not used in the method score.

**Table 2:** Main characteristics of the participants in each study.

Study	Participants (*n*)	Age (Mean ± SD)	ASIA Grade	Level of Injury	Time After Onset Disease (Months)
Gil-Agudo et al. 2012 [7]	*N* = 10. CG: 5, IG: 5	CG: 49.0 ± 6.11, IG: 36.2 ± 10.41	A–B	C5–C8	CG: 5.8, IG: 4.2
Dimbwadyo-Terrer et al. 2013 [11]	*N* = 18. CG: 6, IG: 12	CG: 42.0 ± 13.56, IG: 33.6 ± 14.11	A–D	C5–C8	CG: 3.6, IG. 6.6
D’Addio et al. 2014 [6]	*N* = 30. CG: 15, IG: 15	43.0 ± 18.7	C–D	ND	ND
Dimbwadyo-Terrer et al. 2016 (a) [2]	*N* = 31. CG: 15, IG: 16	CG: 40.2 ± 13.61, IG: 34.5 ± 13.71	A–B	C5–C8	CG: 5.6, IG: 4.3
Dimbwadyo-Terrer et al. 2016 (b) [34]	*N* = 8. CG: 3, IG: 6	CG: 44.2 ± 22.92, IG: 54.3 ± 9.86	A–D	T1–T6	CG: 5, IG: 5.8
Khurana et al. 2017 [35]	*N* = 30. CG: 15, IG: 15	CG: 29.8 ± 7.32, IG: 29.4 ± 7.48	A–B	T6–T12	CG: >6, IG: >6
Prasad et al. 2018 [36]	*N* = 22. CG: 10, IG: 12	CG: 33.9 ± 7.1, IG: 23.7 ± 5.2	A–D	C5–C8	CG: 10.2, IG: 15.2

ASIA: American spinal injury association impairment scale; CG: control group; IG: intervention group; ND: not described.

**Table 3:** Main characteristics of the interventions.

Study	Group Interventions	Intensity	Session Duration	Intervention Duration	Outcome	Measuring Instrument	Results
Gil-Agudo et al. 2012 [7]	CG: Conventional physical therapy, IG: Toyra^®^ VR system	3 times/week	30 min	5 weeks	Upper limb range of motion, motor function and strength. Functional performance	BI, FIM, NHPT, JTT, MI, SCIM	No significant differences were found between groups after intervention, except for JHFT subtest 5 (*p* = 0.00)
Dimbwadyo-Terrer et al. 2013 [11]	CG: Conventional physical therapy, IG: Toyra^®^ VR system	4 times/week	ND	3 weeks	Upper limb range of motion and motor function. Functional performance	FIM, MI, MB, SCIM	No significant differences were found between groups after intervention
D’Addio et al. 2014 [6]	CG: Conventional physical therapy, IG: Nintendo Wii	3 times/week	ND	12 weeks	Posture and balance. Functional performance	BBS, Romberg, posturographic analysis, SCIM	Significant results between groups were found in all parameters: BBS (*p* = 0.02); Romberg (*p* = 0.03); posturography (*p* = 0.03 & *p* = 0.04); SCIM (*p* = 0.02)
Dimbwadyo-Terrer et al. 2016 (a) [2]	CG: Conventional physical therapy, IG: Toyra^®^ VR system	3 times/week	30 min	3 weeks	Upper limb motor function. Functional performance	MMT, MI, FIM, =SCIM, BI	No significant differences were found between groups after intervention. At follow-up only MMT was statistically improved (*p* = 0.04)
Dimbwadyo-Terrer et al. 2016 (b) [34]	CG: Conventional physical therapy, IG: VR system + CiberTouch^™^ data glove	2 times/week	30 min	2 weeks	Upper limb motor function. Functional performance	MB, NHPT, JTT, SCIM.	No significant differences were found between groups after intervention
Khurana et al. 2017 [35]	CG: Conventional physical therapy focused in balance training, IG: Sony Play Station 2 + Eye Toy	5 times/week	45 min	3 weeks	Balance. Functional performance	mFRT, t-shirt test, SCIM	Significant results between groups were found in: mFRT scores (*p* = 0.01); t-shirt test (*p* = 0.01) scores, and in the self-care component of SCIM (*p* = 0.01)
Prasad et al. 2018 [36]	CG: Conventional physical therapy, IG: Nintendo Wii	3 times/week	60 min	2 weeks	Upper limb dexterity and motor function. Functional performance	CUE, BBT, WHOQOL-BREF, SCIM	No significant differences were found between groups after intervention

BBS: Berg balance scale; BBT: box and block test; BI: Barthel index; CG: control group; CUE: capabilities of upper extremity; FIM: functional independence measure; IG: intervention group; JTT: Jebsen Taylor hand function test; MB: muscle balance; mFRT: modified functional reach test; MMT: manual muscle test; MI: motricity index; ND: not described; NHPT: nine hole peg test; SCIM: spinal cord independence measure; VR: virtual reality; WHOQOL-BREF: World Health Organization quality of life-BREF.
